# Patterns of Activity in the Human Frontal and Parietal Cortex Differentiate Large and Small Saccades

**DOI:** 10.3389/fnint.2016.00034

**Published:** 2016-10-27

**Authors:** Marie-Hélène Grosbras

**Affiliations:** ^1^Laboratoire de Neuroscience Cognitive, Aix-Marseille UniversityMarseille, France; ^2^Institute of Neuroscience and Psychology, University of GlasgowGlasgow, UK

**Keywords:** saccades, fMRI, FEF, parietal, eccentricity, spatial exploration

## Abstract

A vast literature indicates that small and large saccades, respectively, subserve different perceptual and cognitive strategies and may rely on different programming modes. While it is well-established that in monkeys’ main oculomotor brain regions small and large eye movements are controlled by segregated neuronal populations, the representation of saccade amplitude in the human brain remains unclear. To address this question we used functional magnetic resonance imaging to scan participants while they performed saccades toward targets at either short (4°) or large (30°) eccentricity. A regional multivoxel pattern analysis reveals that patterns of activity in the frontal eye-field and parietal eye fields discriminate between the execution of large or small saccades. This was not the case in the supplementary eye-fields nor in the inferior precentral cortex. These findings provide the first evidence of a representation of saccadic eye movement size in the fronto-parietal occulomotor circuit. They shed light on the respective roles of the different cortical oculomotor regions with respect to space perception and exploration, as well as on the homology of eye movement control between human and non-human primates.

## Introduction

To explore our environment, we make on average three saccades per second. Most of these saccades are of small amplitude (<10° of visual angle), such as while reading or exploring a picture. Even when scanning the periphery most often we use short eye movements in conjunction with head and or trunk movements. In some other circumstances, however, we also make larger eye movements, like, for example, when checking peripheral objects while driving. The oculomotor apparatus is thus capable of producing saccades of a wide range of amplitudes depending on the context (Yarbus, 1967; Bahill et al., 1975; Leigh and Zee, 1999; Pelz and Canosa, 2001; Borji and Itti, 2014). Furthermore, a number of studies indicate that the size of saccades is closely linked to visual and cognitive processing (Jacobs and O’Regan, 1987; Land et al., 1999; Tatler et al., 2006). Indeed, while small (<10° of visual angle) saccades are more frequent, their proportion, relative to large saccades varies with the task, indicating a functional distinction between the two types of saccades (Tatler et al., 2006). Some authors have proposed that visual exploration with large saccades corresponds to a pre-attentive or “ambient” scanning mode, while small saccades would be associated with a more intensive and detailed “focal” processing (Pelz and Canosa, 2001; Unema et al., 2005; Tatler et al., 2006; Over et al., 2007). This distinction appears to be present very early on in development (as young as 2 years old; Helo et al., 2014), and is also associated with the visual features and duration of successive fixations (Jacobs and O’Regan, 1987). Even for visually guided reflexive saccades (i.e., toward a suddenly appearing visual target), the characteristics of saccades depends upon eccentricity, with smaller saccades having better accuracy than larger saccades, which tend to be hypometric, more variable and faster (Frost and Poppel, 1976; Abrams et al., 1989). The difference between programming small and large saccades might be linked to differences in the organization of the retina, with the ratio between P and M cells decreasing with eccentricity. Related to this, classical ablation (Cowey, 1974) and autoradiographic tracing (Hubel et al., 1975) studies have demonstrated that the central part of the retina is more heavily connected to the geniculo-striate pathway while the peripheral part is more connected with the collicular pathway. Based on these data, Frost and Poppel (1976) have proposed that there exists a qualitative change in programming strategy between small and large saccades with a cut-off at 10–15° of eccentricity.

These observations can be related to the cortical organization of oculomotor control. Neurophysiological and anatomical data obtained in non-human primates indicate that in two of the main cortical regions that control eye movements, namely the frontal and the parietal eye-fields, distinct segregated neuronal populations control small and large saccades, respectively (e.g., Robinson and Fuchs, 1969; Bruce et al., 1985; Goldberg et al., 1986; Stanton et al., 1995; Schall, 1997; Sommer and Wurtz, 2000; Savaki et al., 2010). In contrast, although neurons coding for specific amplitudes have been identified in the supplementary eye-field (SEF), they appear to be intermingled (Schlag and Schlag-Rey, 1987; Russo and Bruce, 1993, 2000).

In the human brain, the homologs of these three eye-fields – frontal, parietal, and supplementary – are well-localized in corresponding parts of the cortex (Lobel et al., 2001; Grosbras and Berthoz, 2003; Amiez and Petrides, 2009). Brain imaging and brain stimulation studies have consistently shown sensitivity to the *direction* of saccades in the frontal eye-field (FEF; e.g., Penfield and Rasmussen, 1952; Grosbras and Paus, 2002; Koyama et al., 2004; Curtis and D’Esposito, 2006; Kastner et al., 2007; Van Pelt et al., 2010; Gallivan et al., 2011; Bender et al., 2013) and in the parietal cortex (e.g., Schluppeck et al., 2005; Medendorp et al., 2006; Knops et al., 2009; Pertzov et al., 2011). Regarding the representation of saccade amplitude, data are sparser. Kimmig et al. (2001) investigated changes in functional magnetic resonance imaging (fMRI) signal linked to saccade size ranging from 4 to 10° in a reflexive visually guided saccade paradigm and did not find any significant effect of size in the oculomotor cortical regions. Petit et al. (2009) found no differences in cortical activity associated with visually guided saccades of 15–21° compared to 3.5° saccades. In a recent study, Leone et al. (2014) adapted a classic retinotopic mapping paradigm to a saccade planning paradigm, asking subjects to make memory-guided saccades toward targets of various directions and eccentricities. They used singular value decomposition of the activity measured during the planning phase and showed that while the topographical organization of the representation of saccade plan was clear in the parietal cortex, it was more variable in the frontal cortex. But the motor execution phase was not analyzed and only eccentricities up to 12° were tested. In fact, most studies only use saccades of small (<5°) or medium (5–15°) amplitudes, maybe because of the restricted visual displays in scanners, precluding addressing the question of different modes of programming depending on the saccade size.

Here, we investigated whether neuronal activity in the different nodes of the human cortical oculomotor network contains information distinguishing small and large saccades, as is the case in non-human primates. We designed an fMRI experiment with short blocks of either large or small saccades. We used multivariate pattern analysis (MVPA) to test whether the pattern of activity amongst the voxels of predefined functional regions can predict the category of saccades being performed (see review in Pereira et al., 2009). We also included a condition consisting of blocks mixing large and small saccades to test for putative adaptation effects. Indeed, in other contexts it has been observed that repeated occurrences of events that share a common neural representation reduce blood-oxygen-level dependent (BOLD) responses relative to presentations of dissimilar features (e.g., Grill-Spector and Malach, 2001; Pellijeff et al., 2006; Dinstein et al., 2007; Kroliczak et al., 2008; Van Pelt et al., 2010). We would expect a region with neuronal activity linked to saccade amplitude to be less active in the case of repeated small or large saccades than in the case of successive saccades of different amplitudes.

## Materials and Methods

### Subjects

Nineteen healthy volunteers (12 females; age: 19–37 years; mean: 23.9 years) with normal or corrected-to-normal vision participated in the study. All gave informed consent and the study was approved by the local ethics committee of the University of Glasgow College of Science and Engineering. Data from two subjects were excluded from the final analysis: one because of poor performance in the task and one because artifact contamination (see Results). Therefore, data is presented for 17 participants (10 females).

### Task Design

**FIGURE 1:**
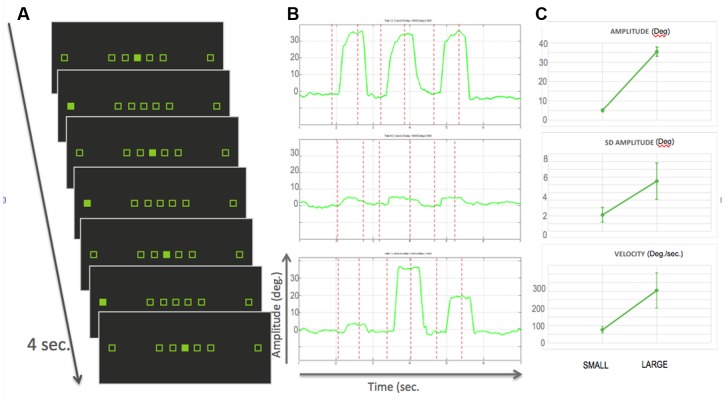
**(A)** Experimental design: the seven squared place-holders stayed displayed on the monitor for the whole experiment. Participants were required to gaze to the filled square, thereby performing small, large or medium size saccades. Each bock consisted of six saccades in 4 seconds. The experiment was an alternation of blocks with either only small, only large saccades or a mix of the three sizes. Two blocks were separated by a variable duration of fixation. **(B)** Example traces of saccades within a run for, from top to bottom, large, small and mixed blocks. Red dotted lines represent the onset of visual targets. **(C)** *Top:* Average amplitude in degrees over the 17 participants for small and large blocks. Error bars represent standard deviation; *Middle:* average standard deviation of the amplitude, as a surrogate measure of spatial accuracy; *Right:* averaged velocity in deg./sec. As expected larger saccades were more variable and faster.

Participants were first trained to perform the visually guided saccade task outside the scanner. The visual display consisted of seven square boxes (0.8°) in the horizontal plane, green on a black background. The centers of the boxes were located at 0, ±5°, ±10°, and ±30° from the center of the screen. The experiment comprised 4-s long mini-blocks, during which a small square filling a box jumped from the central box to a peripheral location and back three times, requiring the observers to make six saccades. After each lateralized saccade, the next one would bring the gaze back to center (i.e., succession of centrifugal and centripetal saccades of same size; see **Figure 1A**). The time interval between two saccades was randomly varied, with an average frequency of 1.5 Hz, and participants were instructed not to make anticipatory movements. Saccades were arranged in mini-blocks of either small (4°), large (30°), or a succession of small, medium, large (4, 10, and 30°) saccades. The main question of interest was the comparison between large and small saccade blocks. The mixed-blocks were introduced in order to test for adaptation effects: we expected no adaptation in the mixed blocks in contrast to the two other categories. That is, we expected higher signal in the mixed blocks. Within a block, all saccades were executed within the same visual hemifield. There were thus six different types of block: small saccades, large saccades or saccades of mixed amplitudes performed either in the left or right hemifield. Each condition was repeated six times during a run. Two consecutive blocks were separated by 6–8 s of central fixation, and the beginning and end of a run started with 8 s of fixation. These served as baseline in our analysis. The order of the blocks within a run was arranged following an m-sequence, such that each condition followed each of the others an equal number of times, thereby optimizing the contrast-to-noise ratio (Buracas and Boynton, 2002). Thirteen subjects performed five runs and four subjects performed six runs.

Visual stimuli and synchronization with brain image acquisition were programmed using Presentation (NeuroBehavioral systems). In the scanner subjects viewed the computer screen through MR-compatible goggles (Nordic Neurolab, screen size 800^∗^ 600 pixels, with a field of view corresponding to about 44^∗^ 20° of visual angle).

### Scanning Parameters and Eye Tracking

Whole-brain echo-planar functional images (echo time 30 ms, TR 2 s, 3 × 3 × 3.3 mm^3^ voxels) were acquired on a 3-Tesla scanner (Siemens Tim Trio) equipped with a 12-channel head coil using integrated parallel imaging techniques (IPAT factor: 2). A high-resolution structural image was acquired with a 3D MPRAGE sequence (1 mm^3^ resolution).

During each functional run, eye movements were recorded using a monocular video-infrared eye tracker (Arrington Viewpoint system, integrated into the goggle display system) with a sampling rate of 60 Hz and a spatial accuracy of about 1° (according to the manufacturer). This required a short calibration completed at the beginning of the scanning session as well as between runs. We analyzed this data semi-automatically using in-house matlab scripts to verify that the participants were performing the task correctly. Eye position data were plotted for each block (see **Figure 1B**), and saccades were identified as horizontal eye deviations larger than 2° from the current fixation position. We classified saccades as small (2–6°), large (>20°), or medium (>6 and <15°). Because we were not looking at a parametric modulation nor at the effect of precision but just a difference of processing linked to large and small saccades, we did not consider spatial accuracy. Blocks for which the sequence of saccades did not correspond to the stimuli (eccentricity and direction) were excluded from the analysis. Following this procedure, we excluded one participant for whom over half of the blocks were incorrect (she made only one saccade or closed her eyes or blinked until the next fixation block). For the remaining 18 participants, 16 performed the task perfectly, for one participant we excluded 18 blocks and for another participant we excluded one full run for which 30/36 blocks were bad.

### Data Analysis

#### Preprocessing and General Linear Model

We analyzed the fMRI data using FSL (FMRIB’s Software Library^1^) and LIBSVM and custom matlab functions for the MVPA. The following pre-processing was applied: motion correction using MCFLIRT (Jenkinson et al., 2002), ensuring that head motion did not exceed 1 mm in any direction; non-brain tissue removal using BET; grand-mean intensity normalization of the entire 4D dataset by a single multiplicative factor; high-pass temporal filtering (Gaussian-weighted least-squares straight line fitting, with sigma = 50.0 s). We used FEAT (FMRI Expert Analysis Tool, part of FSL) Version 5.98 to analyze the time series. For the MVPA, we modeled each individual block as a separate condition and computed one parameter estimate for each block. For the univariate analysis the data was smoothed with a Gaussian kernel 5 mm FWHM. We modeled each of the six block-types as a regressor and its temporal derivative (to account for variations in saccadic reaction time) and performed a voxel-wise general linear model analysis with local autocorrelation correction (Woolrich et al., 2001). We also included six direction head-motion estimates as regressors of non-interest. We computed one parameter estimate per run and averaged across all the runs to obtain one final parameter estimate for each of the six conditions.

The resulting maps of parameter estimates were registered to the high-resolution structural image and then to standard MNI space using linear transformation with nearest-neighbor interpolation (Jenkinson et al., 2002).

#### Regions of Interest (ROIs)

**FIGURE 2:**
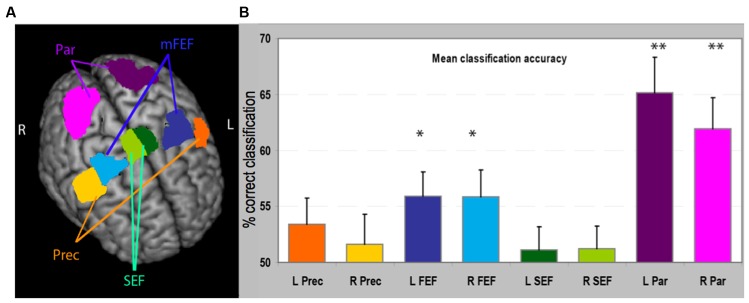
**(A)** Regions of interest (ROIs) used in the analysis reported on a 3D brain template. The stereotaxic coordinates and size of the regions are also reported in **Table 1.** **(B)** Classification accuracy in the MVPA for these eight regions. For each region we have plotted the proportion of times the classifier, trained on independent data, correctly classified BOLD data as related to the execution of small or large saccades. Asterisks denote significantly higher proportion of correct classification than would be expected by chance, i.e., 50% (as determined by a permutation test). mFEF, frontal eye-field; Prec, inferior precentral cortex; Par, parietal cortex region; SEF, supplementary eye-field. ^∗^*p* < 0.05; ^∗∗^*p* < 0.001.

We restricted analyses to four bilateral regions of interest (ROIs). These regions were delineated on the standard MNI-template based on a meta-analysis of 29 visually guided saccade experiments (Grosbras et al., 2005) and included the medial FEF, inferior precentral cortex (Prec.; also referred to sometimes as lateral FEF), SEF (including cingulate eye-field) and parietal (Par.; including intraparietal sulcus and superior parietal lobule and putative homolog of the monkey parietal eye-field). Briefly, the activation likelihood estimation maps represent, at each voxel, the probability of a study on saccadic eye movements reporting an activation focus. They were thresholded at *p* < 0.01 using false discovery rate correction (Laird et al., 2005). Then, within the significant voxels of these maps, we identified four regions bilaterally (see **Figure 2**; **Table 1**): (i) the medial FEF centered on the intersection between the superior frontal and superior precentral sulci, extending dorsally to the surface of the brain; (ii) the inferior precentral eye-field encompassing the most lateral part of the precentral gyrus and extending ventrally to the inferior precentral sulcus near its junction with the inferior frontal sulcus (Lobel et al., 1998); (iii) the SEF centered on the paracentral sulcus (Grosbras et al., 1999), extending ventrally to the cingulate sulcus, dorsally to the surface of the brain and laterally to the first bend of precentral gyrus; anteriorly it was limited by the loop that the cingulate sulcus makes at the level of the anterior commissure (VCA line), which represents the limit with pre-SMA (Picard and Strick, 1996); (iv) the “parietal eye-field” including the region that is believed to be the homolog of the macaque LIP (Sereno et al., 2001; Silver and Kastner, 2009) centered around the middle part of the intraparietal sulcus and extending dorsally to the superior parietal lobule. Overlay with cyto-architectonic maps indicated that this region overlapped partially with BA2 and HIP1 (Eickhoff et al., 2007). ROIs contained between 564 (SEF) and 1043 voxels (parietal region).

**Table 1:** Coordinates of the boundaries of the eight regions.

ROI	X min	X max	Y min	Y max	Z min	Z max	Large vs. Small max Z	Mixed vs. Repeat max Z
Left FEF	-51	-15	-18	9	42	69	3.78	-2.76
Right FEF	24	60	-15	6	42	69	**4.57**	-3.88
Left Prec	-56	-39	-10	12	30	48	3.38	-3.14
Right Prec	40	62	-8	12	26	40	3.93	-2.24
Left SEF	-10	2	-16	4	42	68	3.07	-1.67
Right SEF	2	12	-14	4	42	68	3.32	-1.45
Left Par	-40	-10	-72	-38	48	70	4.01	-3.23
Right Par	14	42	-80	-46	48	74	3.72	-3.00


*Regions are of various shapes; we report the minimum and maximum values for each direction. The last two columns show the maximum *Z*-score from the statistical analysis comparing, voxel-wise, the conditions Large vs. Small (7th column) and Mixed (blocks mixing small, large, and medium saccades) vs. Homogeneous (blocks of Large and Small saccades collapsed). Only values in bold are statistically significant.*

#### Multivoxel Pattern Analysis

We performed one analysis per ROI, using the voxel-wise parameter estimates derived from the general linear model that included one predictor per individual block. For this analysis we considered only the blocks of small or large saccades, as our main hypothesis is that these two types of saccades should be encoded by distinct neuronal populations (it would not be clear what pattern a block of mixed saccades would generate). In each ROI, we trained a linear support vector machine (Chang and Lin, 2011) on the parameter estimate values associated with small or large saccades, collapsing left and right hemifield conditions (thus 12 values per voxel per run for each condition) in all but one run. We then tested how well the classifier could predict the results from the left-out run; that is, how often the model could recognize a block of small saccades as small saccades, and large as large. We used the LIBSVM matlab toolbox, with a regularization parameter of *C* = 1 (as done in the majority of fMRI MVPA studies; e.g., Mourao-Miranda et al., 2005; Pessoa and Padmala, 2007; Eger et al., 2009; Knops et al., 2009; Gallivan et al., 2011). Prior to training, we first removed the mean from individual beta values within each run and ROI (mean-centering) in order to reduce the effect of large, image-wide, signal changes and to deal with potential session effects (Pereira et al., 2009). This was repeated for each run and an average percent correct score was computed for each subject. To assess significance in the group of participants, the percent-correct scores were compared to the theoretical chance level (50%) using exact Student’s *t*-test: we subtracted 50% from each individual averaged accuracy score and computed a *t*-value across participants for each region. Then, to account for the non-normal distribution of accuracy values as well as family wise errors, we used a permutation test to compute an exact threshold for those *t*-values at α = 0.05 at the level of the experiment (i.e., family wise alpha across the eight ROIs). To do so we performed 10000 permutations, randomly reassigning for each subject the sign of the difference between accuracy-score and 50%, before computing a *t*-score across participants for each ROI. For each permutation we extracted the maximum values (*t*-max) across the eight ROIs (Blair and Karniski, 1993). We built a distribution of 10000 *t*-max and compared the observed group *t*-values to this empirical distribution (Blair and Karniski, 1993; Manly, 1997).

Further, in order to explore the effect of the difference in size amongst the ROIs we repeated the analysis using a feature selection approach. We used the GLM result maps of the contrast “all saccades > rest” in each individual run to select only a subset of voxels that showed the highest parameter estimate values in the training set (as in De Martino et al., 2008; Eger et al., 2009). We then repeated the procedure described above for these modified ROIs. We did this for sets ranging from 200 to the maximum number of voxels in the anatomical ROI, stopping at 900 for larger frontal and parietal regions. The family wise permutation testing was performed for each set-size separately, i.e., yielding an exact *t*-value per ROI size.

#### Contrast and Adaptation Analyses

We first contrasted the small and large saccade conditions directly, collapsed across the left and right hemifield. To do so we performed individual general linear model analyses with one regressor per condition (Small; Large; Mixed) using a fixed-effects model (FMRIB’s Local Analysis of Mixed Effects). Then we compared small and large saccade conditions at the group level using a mixed-effect model (FLAME stage 1 and stage 2; Woolrich et al., 2004). Resulting Z-statistics maps were masked by the ROIs and thresholded at *p* < 0.05, corrected for multiple comparisons within the ROIs.

Secondly, we assessed the adaptation effect using the same procedure. We contrasted “mixed-size blocks” with “same-size saccades blocks” (i.e., grouping together large and small saccade blocks). Higher activity in the mixed-blocks would indicate an adaptation effect and thus sensitivity to saccade amplitude.

## Results

### Behavioral Data

As mentioned previously, behavioral data were collected mainly to ensure that the participants performed the task correctly, as, in the present study, we did not look for any continuous parametric modulation of brain activity as a function of saccade sizes. Of the datasets that we retained for the analysis, we could observe that, as expected, saccades of different sizes. Large saccades showed significantly more trial-to-trial variability in their endpoint than the small saccades (*p* < 0.0025, as tested by a *t*-test on the variances computed in individual participants). Large saccades also showed higher mean velocities than small saccades (*p* < 10^-4^) (see **Figure 1C**).

### Multivariate Pattern Analysis

We performed the MVPA to test how well a classifier trained on part of the data, by associating a pattern of activity to a condition, could predict whether new data correspond to the execution of large or small saccades. Accurate prediction would indicate that the activity profile used for training contains significant information about the conditions of interest. The performance of the linear support vector machine classifiers for each ROI is represented in **Figure 2.** The most significant result is an above-chance classification in bilateral parietal areas [left parietal, mean accuracy 65.2%, *t*(16) = 4.88, *p* = 0.000062; right parietal mean accuracy 61.9% *t*(16) = 4.40, *p* = 0.00014]. Classification was also significantly above chance in the bilateral [left FEF mean accuracy 55.9%, *t*(16) = 2.94, *p* = 0.046; right FEF mean accuracy 55.8%, *t*(16) = 2.67, *p* = 0.031].

**FIGURE 3:**
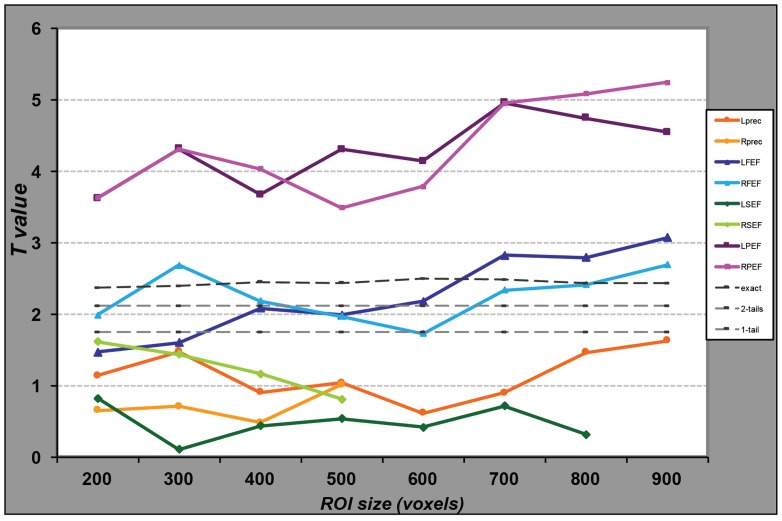
**Performance of the SVM classifiers for regions of different sizes.** For each ROI, represented by a line of distinct color, we selected the voxels presenting the highest parameter estimate values in the univariate analysis and entered them in the MVP analyses. We repeated this procedure for different numbers of voxels, from 200 to the maximum number of voxels in the anatomical ROI, or 900 in case of larger regions. For each MVPA, we computed the proportion of correct classification of data as related to the large or small saccade blocks. We then computed the *t*-value comparing these values to chance (50%) across the group of participants. Light gray dotted lines represent a significant difference from chance at α = 0.05 from a student *t*-test (one or two-tailed); the darker gray line represents the same significance level as determined by data obtained from 10,000 permutations. While the classifier performs significantly above chance for any voxel set size in the parietal regions, for the FEF the classification is significantly above chance only when we include at least 700 voxels in the analysis.

We also tested whether the classification results would depend of the size of the ROI. **Figure 3** represents the observed *t*-values, in the comparison of classification accuracy against chance, when the regional analysis was limited to voxels showing the highest univariate differences between conditions, for various sizes. MVPA in the parietal regions distinguished large from small saccade related activity with high accuracy, even when reducing the region to a small number of voxels. Accuracy performances ranged from 59% correct (for 200 voxels) to 64% (for 700 voxels). Accuracies in the FEF were significantly above chance (accounting for family wise multiple comparisons) only for larger regions (>700 voxels for the left FEF and >800 for the right FEF).

### Contrast Analysis

**FIGURE 4:**
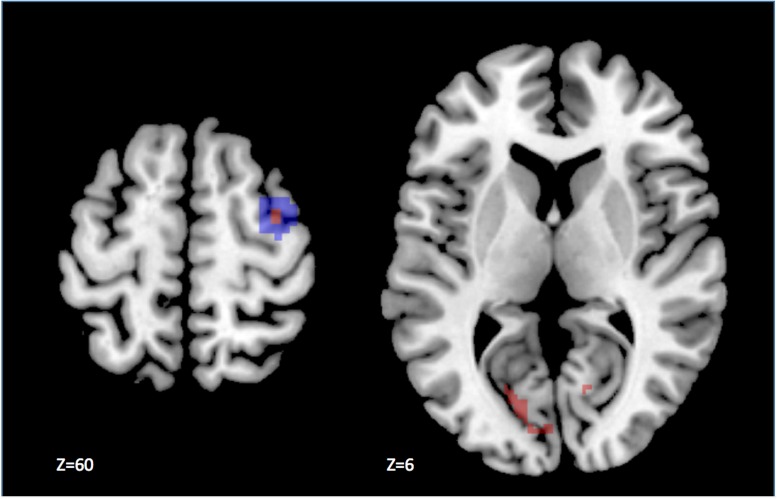
**Results of the univariate analysis contrasting large and small saccade blocks.** The statistical parametric map has been thresholded at *p* < 0.05 corrected for multiple comparisons. The difference in activity is significantly larger during the execution of saccades of large amplitude, as compared to small in a few voxels (in red) located in the dorsal part of the anatomical right FEF ROI (highlighted in blue), displayed on the left panel. In addition, we observed activation outside our ROIs, notably in the visual cortex as shown on the right panel.

We also performed more classical analyses using the general linear model, testing at each voxel the correlation between the fMRI signal and the experimental design. The direct univariate comparison large > small saccade blocks revealed one small cluster of 10 voxels (*p* < 0.05 corrected for multiple comparisons within ROI), located in the dorsal part of the medial FEF region, at the intersection between the superior frontal and superior precentral sulci, in the right hemisphere (standard MNI coordinates *x* = 36, *y* = -4, *z* = 60, *Z*-score = 4.57; see **Figure 4**). Outside the ROIs, we observed higher activity (*p* < 0.001 uncorrected) for large than small saccades in the extrastriate cortex, lingual gyrus, precuneus, cerebellum, and basal ganglia. The reverse contrast, small vs. large saccades, did not reveal any significant activity in or outside the ROIs.

### Adaptation Analysis

This analysis tested, voxel by voxel, whether repeating one condition (i.e., saccades of a specific size) would result in an attenuated signal, which would also be indicative of some coding of saccade amplitude. Contrasting blocks of mixed saccades with blocks of same-amplitude saccades did not reveal any significant activation within the ROIs. Only by lowering the threshold to *p* < 0.01 uncorrected could we observe a very small cluster (5 voxels) within the intraparietal sulcus.

Outside the ROIs, we observed higher activity for mixed than for homogenous blocks in the lingual and parahippocampal gyri, as well as in the posterior cingulate cortex.

## Discussion

Our data show, for the first time in humans, that two cortical eye-fields, namely the eye-movement related part of the parietal cortex and the medial (FEF-*proper*) contain a representation of saccade amplitude. This result complements research that has shown representations of saccade direction in the same regions. It has implications for understanding the organization of the human oculomotor system in comparison with non-human primates.

### Representations for Small and Large Saccades in the Different Cortical Oculomotor Regions

As summarized in the introduction, in both the frontal and parietal eye-fields of the macaque brain, saccades of small and large amplitude are controlled by neurons occupying distinct territories with distinct properties. In the parietal cortex, the highest saccade-related activity, measured with electrophysiological recording, can be observed in the ventral part of the lateral intraparietal sulcus (area LIPv), and this activity is stronger for small saccades (Ben Hamed et al., 2001). Furthermore, using [14C] deoxyglucose imaging, Savaki et al. (2010) have demonstrated a topographical representation of saccade size: the smallest saccades (5°) are represented close to the fundus of the intraparietal sulcus, while larger saccades (10–30°) are represented toward the crown of the sulcus with a gradient that follows the amplitudes. We did not observe a similar topography in the present fMRI data when performing univariate voxel-wise activity: voxels in distinct parts of the IPS did not exhibit significantly different activity for the large and small saccades respectively. In contrast, differences in BOLD signal as a function of saccade amplitude were evident when we considered the patterns of activity within the patch of cortex active during eye movements, including large parts of the intraparietal sulcus and superior lobule. This suggests that some information about saccade size is contained in the activity measured in this entire region and not in specific territories. Further, the accuracy of the pattern classification procedure was above chance when the analysis was repeated for various regional sizes. This shows that even limited regions of the oculomotor parietal cortex are sensitive to saccade size. The significant accuracy for larger regional size could also reflect the robustness of the result regarding putative inclusion of non-informative voxels.

In contrast to the parietal cortex, in the monkey FEF, the continuity of the topographical representation of saccade amplitude is less evident from the published electrophysiological data. Rather, it seems that there are two disjoint subfields associated with saccades of different amplitudes. For instance, microstimulation of the dorsomedial part of the FEF produces large (>25°) saccades, whereas stimulation of neurons in a distinct territory located laterally and ventrally triggers small saccades (Bruce et al., 1985). This is corroborated by anterograde and retrograde tracer studies, which show different patterns of connectivity for the two distinct neuronal populations: small-saccade neurons present more widespread projections to posterior (visual) regions of the brain while large-saccade neuron connectivity is more restricted (Stanton et al., 1995). This is confirmed by an fMRI study of spontaneous inter-regional activity co-variation of signal (resting-state functional connectivity) in five macaque monkeys that showed that the small-saccade FEF exhibits a stronger relation with visual ventral stream regions associated with object processing (Babapoor-Farrokhran et al., 2013).

Here, even when using saccade amplitudes larger than those used in previous human fMRI studies (Kimmig et al., 2001; Petit et al., 2009), our univariate analysis did not reveal any robust effect within the FEF when correcting for multiple comparisons at the whole brain level. Contrary to these previous studies, however, when using small volume correction, we could identify a cluster in the right FEF where activity was higher for large compared to small saccades. This was associated with activation in visual regions. However, the reverse contrast (small vs. large) did not reveal any significant difference in any oculomotor area. One possibility is that large saccades may induce higher visual activity originating from the sweep of the visual display, which was always present. The MVPA demonstrated more convincingly that the functional organization of the overall region is indeed sensitive to saccade size. Although the exact structure of the underlying neuronal activity cannot be directly inferred, this indicates that neuronal activity in the FEF, as measured with BOLD, contains some representation of saccade amplitude.

It has to be noted, however, that the homologies with monkey oculomotor cortical organization are drawn by considering studies using different methodologies, namely microstimulation or single-unit recording vs. fMRI. Therefore, discrepancies could merely reflect the measures afforded by the different methodologies. For instance, Koyama et al. (2004) noted that the FEF identified in macaque monkeys using BOLD fMRI (with visually guided saccade of 5–11°) was larger than the area commonly reported by microstimulation studies. Although sparse, the studies comparing BOLD and electrophysiological measurements in the same animals (review in Ekstrom, 2010; Boynton, 2011) have underlined that the BOLD response is more related to local field potentials than to spike activity and that the concordance between the two methodologies is much stronger in sensory cortices than in higher-level areas. Based on this, Boynton (2011) has proposed that BOLD recording would highlight more feed-back projections and thus top–down processing. One possibility, then, is that if small and large saccade programming relies on different mechanisms balancing feedback and feedforward processing differently, then their contrast will express itself differently in fMRI and electrophysiological studies. That could explain why we observe globally more activity for large than small saccades and nowhere more activity for small than large saccades. Another crucial difference between fMRI and electrophysiological studies is the limited sampling of the latter. Two monkey fMRI studies that have mapped visual representation in the FEF have indeed observed a topographical representation in concordance with microstimulation evidence of visuomotor representation (small eccentricities mapped in ventrolateral locations and large eccentricities in dorsomedial parts). Yet, in addition they identified a cluster of activity linked to large eccentricities in the fundus of the arcuate sulcus (Wardak et al., 2010; Janssens et al., 2014). It is thus possible that, even in the monkey, different patches of large saccade representation exist. This is in line with our results showing more activity for large saccades and a difference between small and large saccades that is revealed when we examine patterns in the entire FEF region.

Altogether these findings point toward distinct mechanisms at play while programming large vs. small saccades. The programming of large saccades might be related to a functional circuit relying more on top–down control. Babapoor-Farrokhran et al. (2013) have suggested, based on functional connectivity studies in macaque, that this circuit, distinct from the one involved in small saccades programming, might be more related to regions involved in reaching movements.

### Differences between Frontal Cortical Oculomotor Regions

Of interest is the difference between the FEF-*proper* and the inferior precentral oculomotor region. Both the univariate contrast and MVPA approaches revealed distinct activity for small and large saccades in the medial FEF (*FEF-proper*) but not in the inferior precentral cortex. Although, it is now well-established that at least two oculomotor regions exist in the frontal cortex of humans (Lobel et al., 2001; Amiez and Petrides, 2009) and non-human primates (Preuss et al., 1996; Fujii et al., 1998), the functional distinction between these regions has not been fully established yet. Koyama et al. (2004), using a visually guided saccade task with fMRI in humans and macaques, reported an absence of asymmetry in the coding of leftward and rightward saccades in both species in the inferior precentral region, contrary to what was observed in the FEF-*proper*. In addition, stimulation in this part of the ventral premotor cortex evokes goal-directed saccades: that is, a given neuron can control saccades of different amplitudes. This, together with our failure to identify differences in activity between small and large saccades, suggests that this inferior precentral region is a homolog to the monkey inferior precentral premotor region (see also Amiez and Petrides, 2009) and that the neural code for saccadic eye movements is different between this region and the FEF-*proper*. The latter would be more closely related to saccade programming and execution while the former may be more involved in saccades goal selection.

As far as the SEF is concerned, the lack of sensitivity to saccades amplitude speaks for a similar coding in the human oculomotor system as in non-human primates.

Thus, taken together, our findings corroborate the similarity of the cortical oculomotor control representation in human and non-human primates. While the inferior precentral and SEF seem to be insensitive to saccade amplitude, both the FEF and the parietal eye-field contain some representation of saccade size although the topographical organization of the underlying neuronal populations might be different to that in monkeys. This ability to distinguish between saccades of large and small amplitude might constitute a neural substrate for different programming modes depending on the landing point eccentricity.

#### Comparing Univariate Contrast, Adaptation, and Multivariate Classifier Techniques

Our design allowed us to compare, within the same experiment and dataset, three analytical approaches. First, the classical univariate contrast analysis did not reveal any robust distinct activity for small and large saccades, with the exception of a small part of the FEF which was more active for large saccades. The adaptation analysis also failed to reveal any effect. MVPA in contrast, showed differences in the patterns of activity associated with large and small saccades respectively. Because the data entered into the classifier was mean-centered, the above-chance classification is not likely to be a by-product of differences in mean activity, but rather due to information linked to the organization of neuronal populations. Similar to what has been described in closely related parietal and frontal regions for attention shifts (Greenberg et al., 2010) and for reference coding of eye movements (Pertzov et al., 2011), our MVPA findings indicate that specifically tuned sub-populations of neurons may be differentially active for saccades of different sizes. The absence of an observed adaptation effect reflects the sensitivity advantage of the MPVA approach over fMRI adaptation, replicating what has been found in visual cortices. For example, Sapountzis et al. (2010) compared the neuronal activity related to orientation gratings in the early visual cortex, first in an event-related adaptation design, and then with MVPA. They found that both methods were in agreement, with high correlation values across different visual areas to distinguish large differences in orientation. For smaller differences in orientation gratings, however, the MVPA still produced above chance classification, while the adaptation analysis failed to show significant selectivity. In addition, it has been argued that adaptation effects arise, at least in part, from contextual factors such as expectation (Summerfield et al., 2008) and attention (Larsson and Smith, 2012). In our paradigm, although the mixed mini-blocks consisted of an alternation of small, medium and large saccades, the sequence was still highly predictable, thereby possibly masking adaptation effects that are linked to higher level processes, which were not the focus of our central question.

## Conclusion

For the first time we have demonstrated that neuronal populations in the human FEF and parietal cortex present a differential pattern of activity for saccades of small vs. large amplitude. We did not observe such a representation in the SEF or inferior precentral oculomotor region, which indicates different roles for the distinct cortical oculomotor regions in visually guided eye movements. This has implications for our understanding of how spatial orientation is represented in the human brain and for drawing homologies with non-human oculomotor systems. Future studies will attempt to assess whether this functional distinction between small and large saccades could be relevant for unraveling the neural substrate of behaviors or cognitive strategies linked to small or large saccades.

## Author Contributions

M-HG initiated the research, collected the data with help of collaborators, analyzed the data and wrote the manuscript.

## Conflict of Interest Statement

The author declares that the research was conducted in the absence of any commercial or financial relationships that could be construed as a potential conflict of interest.

## Footnotes

1 http://fsl.fmrib.ox.ac.uk/fsl/fslwiki/
